# Schädel-Hirn-Trauma

**DOI:** 10.1007/s00115-023-01546-9

**Published:** 2023-09-07

**Authors:** D. Pinggera, P. Geiger, C. Thomé

**Affiliations:** Universitätsklinik für Neurochirurgie, Medizinische Universität Innsbruck, Anichstraße 35, 6020 Innsbruck, Österreich

**Keywords:** Schädel-Hirn-Trauma/Pathophysiologie, Intrakranieller Druck, Neuromonitoring, Posttraumatische Epilepsie, Zerebraler Perfusionsdruck, Brain injuries, traumatic/physiopathology, Intracranial pressure, Neurological monitoring, Epilepsy, post-traumatic, Cerebral perfusion pressure

## Abstract

Das Schädel-Hirn-Trauma (SHT) bezeichnet eine durch äußere Gewalteinwirkung bedingte Schädigung des Gehirnparenchyms. Es verursacht massive individuelle und sozioökonomische Folgen aufgrund der hohen Morbidität und Mortalität. Betroffen sind junge Menschen durch Verkehrs- oder Sportunfälle, aber auch zunehmend alte Menschen durch Stürze im häuslichen Umfeld. Der Begriff SHT umfasst mehrere Krankheitsbilder, die sich in Ursache, Prognose und Therapie unterscheiden. Allen gemein sind jedoch komplexe pathophysiologische Kaskaden, die sich sofort nach dem initialen Trauma entwickeln und über mehrere Tage und Wochen anhalten können. In dieser Phase versucht eine medizinische Behandlung, sei sie chirurgisch oder medikamentös, die Folgen des Primärschadens zu reduzieren. Die Erhaltung eines adäquaten zerebralen Perfusionsdrucks und die Reduktion des Hirndrucks stehen im Vordergrund.

## Abstract (en)

Traumatic brain injury (TBI) describes parenchymal brain damage caused by external forces to the head. It has a massive personal and socioeconomic impact, as it is a disease with high morbidity and mortality. Both young and old people are affected, as a result of traffic or sports accidents as well as due to falls at home. The term TBI encompasses various clinical pictures, differing considerably in cause, prognosis and therapy. What they all have in common is the pathophysiological cascade that develops immediately after the initial trauma and which can persist for several days and weeks. In this phase, medical treatment, whether surgical or pharmacological, attempts to reduce the consequences of the primary damage. The aim is to maintain adequate cerebral perfusion pressure and to reduce intracranial pressure.

## Lernziele

Nach Lektüre dieses Beitrags

- können Sie die verschiedenen Typen von traumatischen intrakraniellen Blutungen erkennen und benennen.
- sind Sie mit den chirurgischen und medikamentösen Therapien des Schädel-Hirn-Traumas vertraut.
- können Sie die die Glasgow Coma Scale anwenden und den Schweregrad eines Schädel-Hirn-Traumas beurteilen.
- können Sie erhöhten intrakraniellen Druck beurteilen und verstehen Sie die zugrunde liegenden pathophysiologischen Veränderungen.
- wissen Sie um die Möglichkeit einer posttraumatischen Epilepsie und kennen die darauf bezogenen Behandlungsempfehlungen.

## Einleitung

Der Begriff des Schädel-Hirn-Traumas (SHT) beschreibt eine vorübergehende oder dauerhafte Schädigung der **zerebralen Funktionen**

Zerebrale Funktionen

als Folge einer **externen Gewalteinwirkung**

Externe Gewalteinwirkung.

Da der Primärschaden nicht reversibel ist, zielt die Behandlung des SHT auf die sekundären Folgen des Traumas. Die verschiedenen Verletzungsfolgen unterscheiden sich teilweise erheblich. Der vorliegende Beitrag bietet einen Überblick über die verschiedenen Verletzungsfolgen und deren Behandlung.

## Epidemiologie und Altersstruktur

Das SHT ist weltweit eine der **häufigsten Todesursachen**

Häufigste Todesursachen

mit erheblichen gesundheitlichen und sozioökonomischen Folgen, die sich oft erst verzögert manifestieren. Schätzungsweise leben in den USA etwa 5,3 Mio. Menschen und in der Europäischen Union etwa 7,7 Mio. Menschen mit den Folgen eines SHT. In Deutschland und Österreich beträgt die jährliche Inzidenz des SHT etwas über 300 pro 100.000 Einwohner, was vergleichbar mit anderen europäischen Ländern ist [1, 2]. In Deutschland wurden 2021 beispielsweise etwas mehr als 350.000 Patient:innen aufgrund eines SHT jedweden Schweregrads vollstationär behandelt [3]. Es wird geschätzt, dass sich die **Kosten**

Kosten

für Behandlung und Langzeitfolgen auf mehr als 2,5 Mrd. € belaufen [4]. Noch bedeutsamer ist die Tatsache, dass in der Altersgruppe der 29- bis 45-Jährigen das SHT zu den Hauptursachen für Tod oder lebenslange Behinderung zählt und hier primär Männer betrifft [5].

Den überwiegenden Anteil der Verletzungen machen **leichte SHT**

Leichtes SHT

aus, nur in etwa 3 % der Fälle liegt ein mittelschweres, in 5 % ein schweres SHT vor [4, 6]. Die häufigsten Ursachen sind **Stürze**

Stürze,

gefolgt von Verkehrsunfällen, Sportverletzungen und Arbeitsunfällen [1, 7]. Die Epidemiologie des SHT befindet sich jedoch im Wandel, das Altersspektrum verschiebt sich zusehends. Neben einem Altersgipfel in der Gruppe der Kleinkinder und der 20- bis 30-Jährigen kommt es aufgrund einer erhöhten Alltagsaktivität sowie wegen Stürzen im häuslichen Umfeld zu einer Zunahme der Inzidenz bei **älteren Patient:innen**

Ältere Patienten

[7]. Eine multizentrische Studie aus dem Jahr 2021 an sieben deutschen Traumazentren zeigte, dass ein Viertel der Verletzten 75 Jahre oder älter war. In dieser Altersgruppe gab es keinen signifikanten Unterschied mehr zwischen männlichen und weiblichen Patienten [8, 9].

### Merke

Das SHT hat drei Altersgipfel mit einer Zunahme der älteren Patienten in den letzten Jahren.

## Einteilung und Klinik

Der Begriff SHT umfasst ein **großes Spektrum**

Großes Spektrum

an Krankheitsbildern. Diese können hinsichtlich des Verletzungsmechanismus, des weiteren Verlaufs, der Symptomatik und im Zuge dessen hinsichtlich der Therapieauswahl und Prognose unterschieden werden. Allgemein versteht man unter einem SHT die Einwirkung einer **externen Kraft**

Externe Kraft

auf den Schädel und das darunterliegende Gewebe. In der Folge können **verschiedene Verletzungsmuster**

Verschiedene Verletzungsmuster

entstehen, die verschiedene anatomische Strukturen betreffen können:

- Schädelknochen
- Dura
- Hirnparenchym
- Arterielle und venöse Gefäße
- Liquorräume

Die Schädigungen können ohne nachweisbare Folgen bleiben oder mit erkennbaren vorübergehenden oder bleibenden Schäden der neurologischen, kognitiven oder psychosozialen Funktionen einhergehen [10]. Des Weiteren kann zwischen einem gedeckten oder offenen SHT differenziert werden, wobei es bei letzterem durch eine Verletzung der Hirnhäute zur Eröffnung der Liquorräume mit intrakraniellem Lufteintritt (**Pneumozephalus**

Pneumozephalus)

kommt.

Die Schwere eines SHT wird in der Regel anhand der Glasgow Coma Scale (**GCS**

GCS)

bewertet, welche die Augenöffnung und damit die Bewusstseinslage, verbale Reaktionen und motorische Fähigkeiten der Patient:innen berücksichtigt (Tab. 1; [11]). Daraus resultiert eine Einteilung in drei Gruppen:

- Leichtes SHT (GCS 13–15)
- Moderates SHT (GCS 9–12)
- Schweres SHT (GCS 3–8)

**Table 1:** 

Punkte	Augen öffnen	Verbale Reaktion	Motorische Reaktion
6	–	–	Befolgt Aufforderungen
5	–	Orientiert	Gezielte Schmerzabwehr
4	Spontan offen	Verwirrt, desorientiert	Ungezielte Schmerzabwehr
3	Auf Ansprache	Unzusammenhängende Worte	Beugesynergismen auf Schmerz
2	Auf Schmerz	Unverständliche Laute	Strecksynergismen auf Schmerz
1	Keine Reaktion	Keine Reaktion	Keine Reaktion

Der GCS-Score sollte bei der **ersten Beurteilung**

Erste Beurteilung

durch den Notarzt/die Notärztin oder spätestens bei der Aufnahme in das Traumazentrum erhoben werden. Erwähnenswerte Limitation der GCS im klinischen Alltag sind die **prähospitale Analgosedierung**

Prähospitale Analgosedierung

und der Einfluss zentral wirksamer Substanzen sowie die Beurteilung kindlicher Traumata. Idealerweise sollte die Einstufung des GCS-Scores nach Korrektur von Hypoxie und Hypotonie und vor Applikation bzw. nach Metabolisierung von Hypnotika, Sedativa, Analgetika und Relaxanzien erfolgen. Zudem gilt es, eine **Intoxikation**

Intoxikation

mit Genuss- oder Suchtmitteln (beispielsweise Alkohol) zu berücksichtigen. Weitere Limitationen sind die **Interrater-Reliabilität**

Interrater-Reliabilität

und die Beeinflussung durch weitere Verletzungsmuster und Therapiemaßnahmen. Die GCS beinhaltet keine Beurteilung der Pupillenweite und Lichtreaktion und berücksichtigt keine Hirnstammreflexe [12, 13].

### Merke

Ein SHT wird mithilfe der GCS in drei Schweregrade eingeteilt. Dabei werden die besten motorischen und verbalen Reaktionen sowie das Augenöffnen berücksichtigt.

## Krankheitsbilder

### Akutes Subduralhämatom

Das akute Subduralhämatom (aSDH) beschreibt eine Blutung zwischen **Dura mater**

Dura mater

und **Arachnoidea**

Arachnoidea,

die Blutungsquelle kann dabei arteriell oder venös sein. Meist kommt es durch das Trauma zum Zerreißen von Brückenvenen und somit zur **Hämatomvergrößerung**

Hämatomvergrößerung.

Das aSDH tritt in 12–29 % der schweren SHT-Fälle auf. Betrachtet man alle Fälle, präsentieren sich etwa 11 % der Patient:innen mit einem aSDH [14, 15, 16]. Bildgebend zeigt sich in der kranialen Computertomographie (cCT) eine zum Hirnparenchym konkave, hyperdense Läsion, die nicht von den knöchernen Suturen begrenzt wird und sich meist langstreckig über den Kortex erstreckt.

Eine Indikation zur Operation ist laut Brain Trauma Foundation (BTF) gegeben, wenn die Blutung eine Dicke von mehr als 10 mm hat oder eine Mittellinienverschiebung größer als 5 mm in der cCT erkennbar ist, unabhängig vom GCS-Score. Darüber hinaus wird bei Patienten mit einem GCS-Score < 9 eine Operation empfohlen, wenn der GCS-Score vom Zeitpunkt der Verletzung bis zur Krankenhausaufnahme um 2 Punkte gesunken ist, wenn der Patient asymmetrische oder starre und erweiterte Pupillen aufweist und/oder wenn der intrakranielle Druck („intracranial pressure“ [ICP]) konstant über 20 mm Hg beträgt, unabhängig von der Dicke des Hämatoms sowie der Mittellinienverschiebung [17]. Der chirurgische Eingriff sollte möglichst zeitnah nach dem Trauma erfolgen. Methode der Wahl ist eine **Kraniotomie**

Kraniotomie

bis hin zur **dekompressiven Kraniektomie**

Dekompressive Kraniektomie

(Abb. 1).

**Figure 1:**
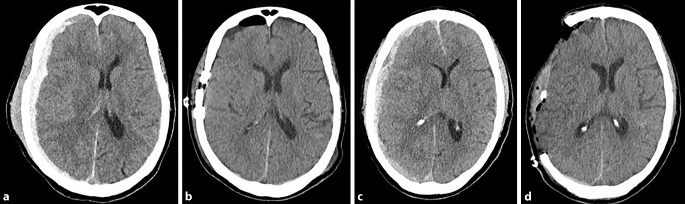

#### Merke

Das aSDH stellt in der Regel einen Notfall dar, der einer chirurgischen Therapie bedarf. Es ist verbunden mit einer hohen Mortalität und Morbidität.

### Chronisches Subduralhämatom

Das chronische Subduralhämatom (cSDH) ist ähnlich dem aSDH durch eine Blutung zwischen Arachnoidea und Dura mater charakterisiert, wobei es sich jedoch nicht um frisches Blut handelt, sondern meist lediglich um **verflüssigte Blutabbauprodukte**

Verflüssigte Blutabbauprodukte.

Häufig ist die Ursache ein **Bagatelltrauma**

Bagatelltrauma,

das mindestens 3 Wochen zurückliegt [15, 18]. In nur etwa der Hälfte der Fälle lässt sich ein Sturzereignis in der Vorgeschichte erheben, in 30–50 % der Fälle ist keine direkte Kopfverletzung eruierbar. **Ältere Personen**

Ältere Personen

entwickeln mit höherer Wahrscheinlichkeit ein cSDH. Bei den betroffenen Patient:innen handelt es sich mehrheitlich um männliche Personen und typischerweise weisen diese ein höheres Lebensalter auf, im Durchschnitt über 70 Jahre [18, 19, 20]. Auch der zunehmende Einsatz einer **gerinnungsbeeinflussenden Medikation**

Gerinnungsbeeinflussende Medikation

scheint die Entwicklung eines cSDH zu begünstigen [21].

Bildgebend zeigt sich in der cCT eine zum Hirnparenchym konkave, überwiegend hypodense Läsion, die nicht von den knöchernen Suturen begrenzt wird und sich regelhaft über die gesamte Konvexität ausbreitet. In etwa 20 % der Fälle sind **bilaterale cSDH**

**Figure 2:**
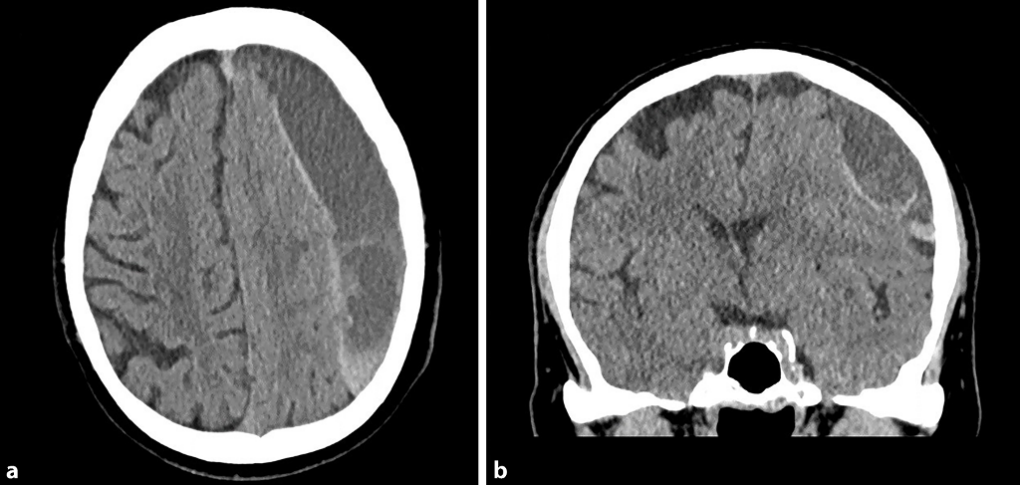

Bilaterales cSDH

zu verzeichnen (Abb. 2 und 3; [22]).

**Figure 3:**
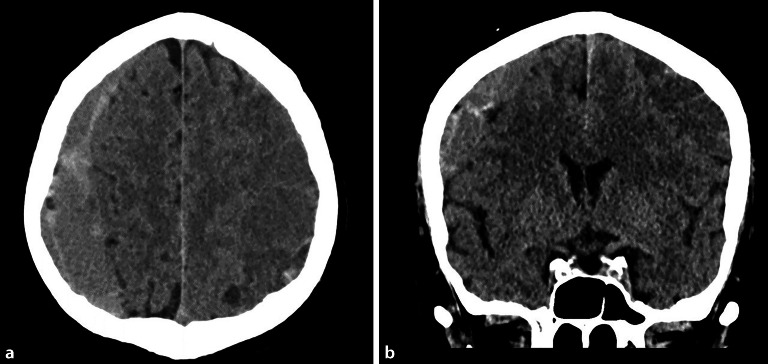

Klinisch auffällige Patient:innen oder jene mit radiologischem Nachweis einer **zerebralen Parenchymkompression**

Zerebrale Parenchymkompression

sollten, unter Berücksichtigung des individuellen Risikos, einer **operativen Intervention**

Operative Intervention

unterzogen werden. Es stehen einige operative Techniken zur Verfügung, wobei die **Bohrlochtrepanation**

Bohrlochtrepanation

das Standardverfahren darstellt. In bis zu einem Drittel der Fälle kommt es allerdings zu einem **Rezidivhämatom**

Rezidivhämatom

[23]. Bei asymptomatischen Patienten müssen die chirurgischen Risiken gegen die des nichtoperativen natürlichen Verlaufs des cSDH abgewogen werden. Ein cSDH kann sich nämlich auch über einen Zeitraum von mehreren Wochen spontan zurückbilden. Alternativ zur chirurgischen Therapie wird in den letzten Jahren auch die **Embolisation**

Embolisation

der A. meningea media als **endovaskuläre Alternative**

Endovaskuläre Alternative

propagiert, da diese die Rezidivhäufigkeit reduziert [24].

#### Merke

Das cSDH entsteht meist verzögert nach Bagatelltraumata. Die Patient:innen präsentieren sich oft mit einer sehr milden Symptomatik.

### Intrazerebrale Blutung

Eine traumatische intrazerebrale Blutung lässt sich bei 13–35 % der Patient:innen mit einem SHT diagnostizieren [25]. Meist kommt es durch eine primäre Schädigung von Gefäßen und/oder des Hirnparenchyms zu einer traumatischen intrazerebralen Blutung oder zu **Kontusionsblutungen**

Kontusionsblutungen,

der Übergang ist hierbei fließend.

**Figure 4:**
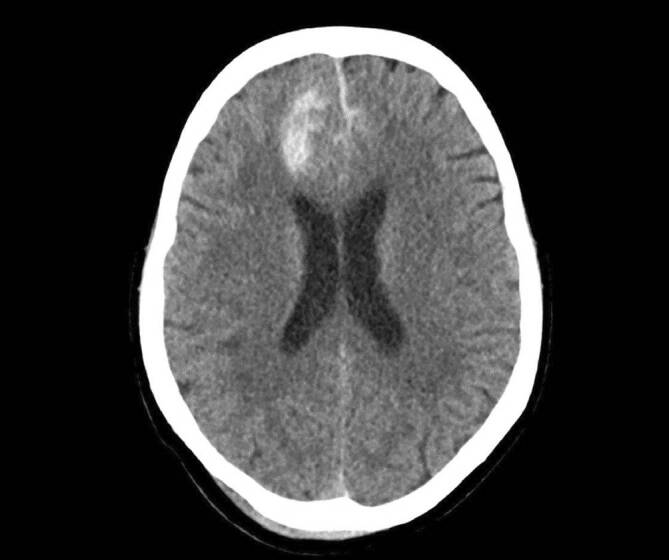

In der cCT können sowohl hyperdense als auch hypodense Areale erkennbar sein. Eine Kontusionsblutung kann sich in multiplen kleinen intrazerebralen Läsionen bis hin zu großen raumfordernden Blutungen manifestieren und in manchen Fällen kann im Rahmen einer Gefäßdarstellung sogar eine aktive Blutung im Sinne eines Kontrastmittelaustritts sichtbar sein (Abb. 4).

Laut BTF besteht die Indikation für eine operative Intervention bei **parenchymalen Massenläsionen**

Parenchymale Massenläsionen

mit einem Volumen von mehr als 50 cm^3^, bei Anzeichen einer fortschreitenden **neurologischen Verschlechterung**

Neurologische Verschlechterung,

bei therapierefraktärer **intrakranieller Hypertension**

Intrakranielle Hypertension

oder bei Patient:innen mit GCS-Scores von 6 bis 8, die frontale oder temporale Kontusionen mit einem Volumen von mehr als 20 cm^3^ und eine Mittellinienverschiebung von mindestens 5 mm aufweisen [17].

### Epiduralhämatom

Das Epiduralhämatom (EDH) befindet sich zwischen Schädelknochen und Dura mater. In den meisten Fällen handelt es sich um eine **arterielle Blutung**

Arterielle Blutung

aus der A. meningea media oder ihren Ästen (> 80 %; [26, 27]). In den restlichen Fällen resultiert das EDH aus einer blutenden Fraktur oder aus der Verletzung eines venösen Sinus.

Typischerweise manifestiert sich ein EDH mit **Bewusstlosigkeit**

Bewusstlosigkeit.

Das sogenannte „luzide Intervall“ tritt nur in seltenen Fällen auf. Verzögert kann eine **rapide Verschlechterung**

Rapide Verschlechterung

eintreten, da durch die Blutung der ICP rasch steigt und es zur Einklemmungssymptomatik kommt, zur starren Pupillenerweiterung und zum Atemstillstand.

In der Neurochirurgie stellt das Epiduralhämatom einen **absoluten Notfall**

Absoluter Notfall

dar. Es tritt bei etwa 5–8 % aller SHT-Verletzungen auf und geht mit einer Mortalitätsrate von 10 % einher. Häufiger sind jüngere Personen betroffen, bei denen sich die Dura leichter vom Knochen ablöst [26, 28]. Wenn die klinischen und bildgebenden Kriterien erfüllt sind, ist die **schnellstmögliche Entleerung**

Schnellstmögliche Entleerung

des EDH die effektivste und wichtigste Therapieoption.

**Figure 5:**
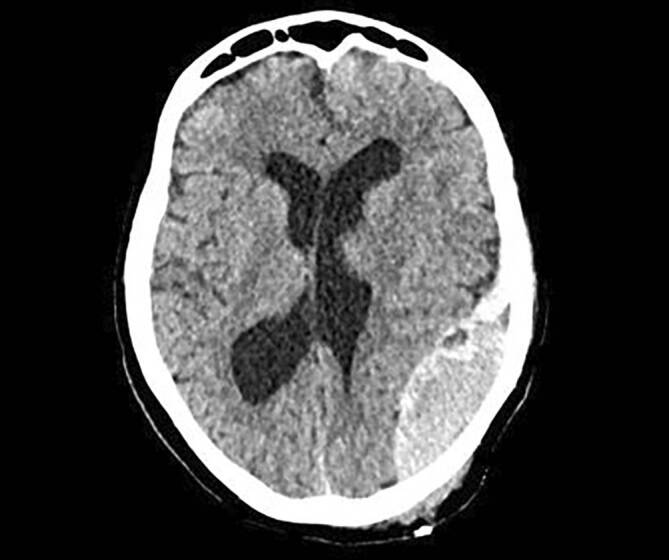

Charakteristischerweise zeigt sich in der Bildgebung eine bikonvexe, kalottenanliegende und hyperdense Läsion (Abb. 5).

Nach den aktuellen Leitlinien der BTF sollte unabhängig vom GCS-Score ein EDH größer als 30 cm^3^ chirurgisch evakuiert werden. Liegen kleinere Läsionen mit einem GCS-Score über 8 ohne neurologische Defizite vor, kann eine **konservative Therapie**

Konservative Therapie

mit engmaschiger Überwachung mittels cCT und genauer neurologischer Beobachtung in einem neurochirurgischen Zentrum ausreichend sein [17, 29].

#### Merke

EDH sind meist temporal und durch eine Ruptur der A. meningea media verursacht. Die chirurgische Hämatomentleerung hat bei symptomatischen Fällen den höchsten Stellenwert.

### Diffuse axonale Schädigung

Die Axone in der **weißen Substanz**

Weiße Substanz

des Gehirns scheinen besonders anfällig für Verletzungen durch mechanische Belastung während eines SHT zu sein [30]. Das Läsionsmuster der diffusen axonalen Schädigung („diffuse axonal injury“ [DAI]) tritt vor allem nach Dezelerations- und Akzelerationstraumata des Gehirns auf. Eine diffuse axonale Schädigung kann bei jedem Schweregrad eines SHT auftreten, so auch bei einem leichten SHT. Im Falle einer Bewusstlosigkeit ist die Prognose deutlich schlechter [31, 32].

Die Symptomatik der betroffenen Person kann sich je nach Ausmaß der Verletzung durch einen **akuten Bewusstseinsverlust**

Akuter Bewusstseinsverlust

oder durch **Verwirrtheit**

Verwirrtheit

äußern und kann als Koma und/oder kognitive Dysfunktion fortbestehen. Initial zeigt sich oft eine Disproportionalität zwischen den klinischen Symptomen und den radiologischen Befunden.

In der cCT können sich kleine punktförmige hyperdense Läsionen zeigen, während sich die Magnetresonanztomographie (MRT) für eine genauere Beurteilung eignet. Dabei präsentiert sich die DAI in Form **petechialer Blutungen**

**Figure 6:**
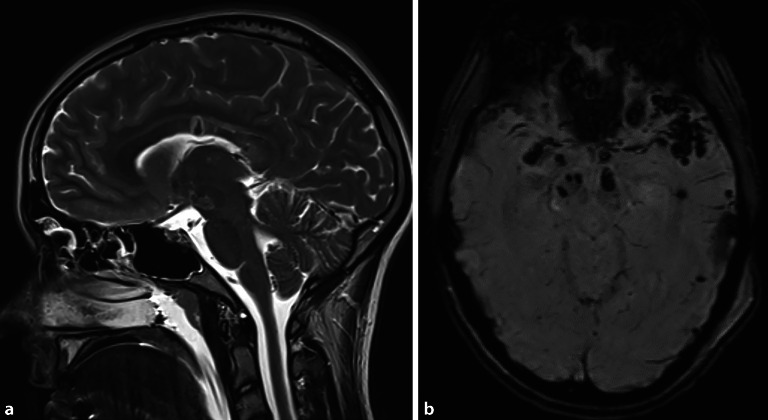

Petechiale Blutungen,

vor allem im Bereich des Hirnstamms, des Balkens sowie entlang der Rinden-Marklager-Grenze (Abb. 6).

Eine spezifische Behandlung gibt es nicht, sodass eine Therapie an die bestehende Symptomatik und den Hirndruck angepasst werden sollte bzw. von anderen Verletzungsfolgen abhängt.

#### Merke

Eine DAI entsteht primär durch ein Schertrauma und ist eine Hauptursache für Bewusstlosigkeit und schlechtes Outcome.

### Traumatische Subarachnoidalblutung

Die traumatische Subarachnoidalblutung („traumatic subarachnoid hemorrhage“ [tSAH]) stellt einen **häufigen Begleitbefund**

Häufiger Begleitbefund

bei moderaten und schweren SHT-Fällen dar, die Blutung tritt bei 33–60 % der betroffenen Patient:innen auf [33]. Durch ein Trauma können **kortikale Gefäße**

**Figure 7:**
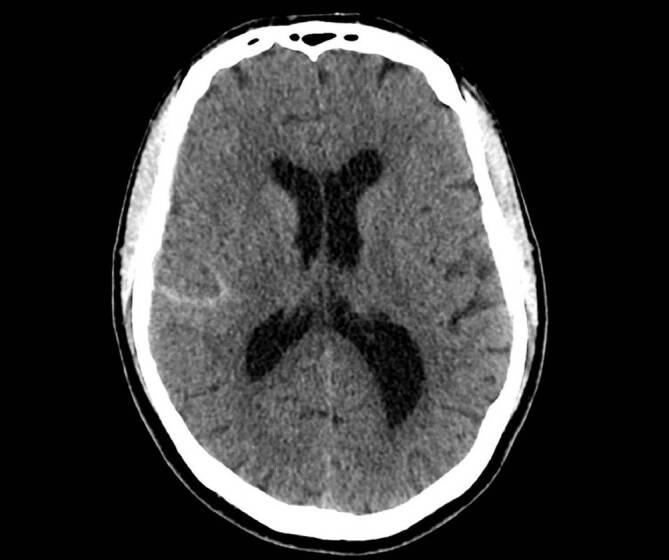

Kortikale Gefäße

rupturieren und es kommt zur Einblutung in den angrenzenden Subarachnoidalraum. Mittel der Wahl zur Diagnosesicherung ist die cCT. Die Läsion zeigt sich dort hyperdens (Abb. 7).

Prinzipiell richtet sich die Therapie bei tSAH nach dem Schweregrad des SHT sowie den Begleitverletzungen. Prognostisch ist besonders eine schwerere Form der tSAH als ungünstig zu werten, auch aufgrund der Gefahr eines **verzögerten Vasospasmus**

Verzögerter Vasospasmus.

### Frakturen

Der primäre Zweck des knöchernen Schädels besteht darin, das Gehirn zu schützen. Wenn sich die Energie auf den Knochen entlädt und zu einer Fraktur führt, wird die Gewalt, die auf das Gehirn übertragen wird, verringert. Es gibt generell zwei Arten von Frakturen:

- Frakturen an der Schädelbasis
- Frakturen an der Schädelkalotte

Alle Frakturen können in der Regel problemlos heilen, es besteht aber die Möglichkeit, dass an der Bruchstelle Blutungen auftreten, die Druck auf das Gehirn ausüben. Die **scharfen Bruchkanten**

Scharfe Bruchkanten

können kleine Blutgefäße zerreißen, was zu einem **epiduralen Hämatom**

Epidurales Hämatom

führen kann; insbesondere im Bereich der Schädelbasis kann dies aber auch Schädigungen der **Hirnnerven**

Hirnnerven

bedingen, beispielsweise einhergehend mit Schwerhörigkeit durch Beeinträchtigung des Hörnervs oder mit Gesichtslähmungen. Verletzungen an der vorderen Schädelbasis können zu einer Verbindung zwischen der Außenwelt und dem Gehirn führen, was unter anderem zu einem Ausfluss von Liquor führen kann. Diese sogenannte **traumatische Liquorrhö**

Traumatische Liquorrhö

tritt bei 10–30 % aller Schädelbasisfrakturen auf [34]. Wenn der Austritt von Hirnwasser nach etwa einer Woche nicht sistiert, ist eine invasive Behandlung erforderlich, entweder im Sinne einer **lumbalen Drainage**

Lumbale Drainage

oder einer **operativen Abdichtung**

Operative Abdichtung.

Ist der Knochen um mehr als **Kalottenbreite**

Kalottenbreite

eingedrückt und kommt es zu einer Impression des Knochens (**Impressionsfraktur**

Impressionsfraktur),

besteht eine Operationsindikation. Eine konservative Behandlung ist möglich, wenn

- keine klinischen oder bildgebenden Anzeichen für eine Durchdringung der Dura,
- kein signifikantes intrakranielles Hämatom,
- keine Eindrückung von mehr als 1 cm,
- keine Beteiligung des Sinus frontalis,
- keine grobe kosmetische Deformierung,
- keine Wundinfektion,
- kein Pneumozephalus und
- keine grobe Wundkontamination

vorliegen [35].

### Chronische traumatische Enzephalopathie

Eine Folge von repetitiven, meist leichten SHT ist die chronische traumatische Enzephalopathie (CTE). Durch **wiederholte Traumata**

Wiederholte Traumata

kommt es zu einer schrittweisen Zerstörung von Nervenzellen und einer ungewöhnlichen Anreicherung des **Tau-Proteins**

Tau-Protein,

ähnlich wie bei der Alzheimer-Erkrankung und anderen neurodegenerativen Erkrankungen. Dies führt zu Veränderungen der kognitiven Fähigkeiten sowie zu Verhaltensauffälligkeiten. Eine spezifische Behandlungsmöglichkeit für eine vermutete CTE gibt es gegenwärtig nicht, man kann nur im Sinne einer **Primärprävention**

Primärprävention

aktiv werden [36, 37].

## Diagnostik

### Bildgebung

Die **cCT**

cCT

ist die meistgenutzte Bildgebungsmodalität in der **Akutphase**

Akutphase

nach Kopfverletzungen. Sie kann Blutungen, parenchymale Verletzungen und knöcherne Schädelverletzungen detektieren und so richtungsweisend sein bei der Entscheidung, ob eine chirurgische Intervention notwendig ist; auch für **postoperative Verlaufskontrollen**

Postoperative Verlaufskontrollen

kann sie herangezogen werden [38, 39, 40]. In der Akutphase ist zudem oft eine Gefäßdarstellung hilfreich, um traumatische Gefäßverletzungen zu detektieren, aber auch um in manchen Fällen einen frühen Perfusionsstopp zu erkennen. Aufgrund der **begrenzten Weichteilauflösung**

Begrenzte Weichteilauflösung

lassen sich kleinere Parenchymdefekte jedoch nicht gut darstellen, sodass für die Detaildarstellung die **MRT**

MRT

das Mittel der Wahl ist. In der Akutphase ist die MRT unter anderem nützlich, um den Schweregrad des akuten Gewebeschadens zu ermitteln; sie kann somit bei der **Prognosebestimmung**

Prognosebestimmung

helfen [41, 42, 43, 44]. Nachteile der MRT – vor allem in der frühen Phase – sind jedoch die **lange Untersuchungsdauer**

Lange Untersuchungsdauer,

die erschwerte Möglichkeit der kontinuierlichen Überwachung während der Untersuchung sowie potenzielle Einschränkungen durch liegende Neuromonitoring-Sonden.

### Biomarker

Als diagnostisches Werkzeug werden auch Biomarker aus dem Serum verwendet, die einige Erfolge bei der Vorhersage der Positivität von CT-Scans beim leichten SHT zeigen. So ist der Biomarker **S100B**

S100B

in Europa weit verbreitet; der **GFAP/UCH-L1-Tandemtest**

GFAP/UCH-L1-Tandemtest

ist in den USA zugelassen [45]. Trotz beeindruckender Sensitivitätswerte weisen diese Tests jedoch keine hohe Spezifität auf. Die Korrelationen zwischen den Biomarkerwerten im Blut und dem Schweregrad eines SHT waren bisher enttäuschend.

#### Merke

Die cCT ist das Mittel der Wahl zur ersten Beurteilung des SHT, da sie Frakturen und Blutungen unmittelbar und sensitiv darstellt.

## Pathophysiologie

Das SHT ist nicht als einzeitiges Ereignis zu werten, sondern vielmehr als eine **Kaskade**

Kaskade

pathophysiologischer Veränderungen, die nach dem Primärschaden beginnt. Initial gehen Neurone zugrunde; die Membrandurchlässigkeit für Ionen und auch die Oxygenierung des Hirngewebes sind gestört. Diese Mechanismen führen innerhalb weniger Stunden zur Störung der zerebralen Durchblutung mit konsekutiver Bewusstseinstrübung [46, 47]. Des Weiteren kann es zur Ausbildung eines **posttraumatischen Ödems**

Posttraumatisches Ödem

kommen, das zu einer Erhöhung des ICP führt, was wiederum eine Reduktion des zerebralen Perfusionsdrucks („cerebral perfusion pressure“ [CPP]) bis zur Entstehung einer fokalen bzw. globalen Ischämie bedingt. Des Weiteren kommt es in dieser Phase durch Störung der Membrankanäle und Ionenpumpen auch zu einem Ungleichgewicht ionischer Gradienten mit kolloidosmotisch bedingter zellulärer Schwellung. Ischämie, vasogenes und zelluläres Ödem können zügig in einen **Circulus vitiosus**

Circulus vitiosus

münden. In der Folge kommt es zu weiteren massiven Veränderungen, die unter anderem mit Entzündungsreaktionen im Sinne einer **Neuroinflammation**

Neuroinflammation

verbunden sind [48].

Die **Spätphase**

Spätphase

tritt Tage bis Wochen nach dem SHT ein und ist gekennzeichnet durch einen **chronischen Entzündungsprozess**

Chronischer Entzündungsprozess.

Der eigentliche Zweck der Neuroinflammation ist die Reparatur des entstandenen Schadens. Dadurch wird GAP43, ein Marker für axonales Wachstum, exprimiert, infolgedessen Axone in die Umgebung sprießen und neuronale Netzwerke neu organisiert werden.

Insgesamt ist die Aktivität der **Mikroglia**

Mikroglia

und der **infiltrierenden Makrophagen**

Infiltrierende Makrophagen

entscheidend für die Neuroinflammation in den Tagen und Wochen nach einem SHT. Obwohl schon in mehreren experimentellen Modellen gezeigt wurde, dass eine gezielte Beeinflussung der Neuroinflammation den biologischen Prozess der Schädigung verändern kann, hat sich leider noch keine pharmakologische Strategie zur Verringerung der Entzündung in klinischen Studien bewährt [49].

### Merke

Ein posttraumatisches Ödem führt zu einer Erhöhung des ICP.

### Autoregulation, zerebraler Perfusionsdruck

Durch Autoregulation wird beim Gesunden ein ausreichender **zerebraler Blutfluss**

Zerebraler Blutfluss

über einen weiten Bereich des mittleren arteriellen Drucks (MAP; 50–150 mm Hg) gewährleistet. Bei etwa einem Drittel der Patient:innen mit schwerem SHT zeigt sich die Autoregulation des Gehirns jedoch gestört. Bei den Betroffenen kann ein **erhöhter MAP**

Erhöhter MAP

mit einem erhöhten Blutvolumen und einer Stauung im Gehirn verbunden sein, was zu einem erhöhten ICP führt, während ein **verringerter MAP**

Verringerter MAP

mit Minderdurchblutung und Ischämie einhergehen kann [50].

Da der zerebrale Blutfluss bettseitig schwer zu eruieren ist, wird alternativ der CPP herangezogen, der sich folgendermaßen berechnet:

- CPP = MAP − ICP

Um Überleben und funktionelles Outcome zu verbessern und günstige Ergebnisse zu erzielen, wird ein CPP von 60 bis 70 mm Hg empfohlen. Bemühungen zur Optimierung des CPP sollten sich zunächst auf die Behandlung eines erhöhten ICP konzentrieren.

Bei moderatem oder schwerem SHT hat sich auch die kontinuierliche Überwachung der **zerebralen Oxymetrie**

Zerebrale Oxymetrie

oder **Druckreaktivität**

Druckreaktivität

etabliert. Auf diese Weise kann die Funktion der Autoregulation bestimmt und ein patientenindividueller CPP angestrebt werden. In diesem Sinne muss auch auf die intensivmedizinische Prävention einer **Hypotonie**

Hypotonie

hingewiesen werden. Es wird ein systolischer Blutdruck von ≥ 100 mm Hg bei Patienten im Alter von 50 bis 69 Jahren und von ≥ 110 mm Hg bei Patienten im Alter von 15 bis 49 oder > 70 Jahren empfohlen [17, 51].

#### Merke

Der CPP sollte über 60 mm Hg liegen, um eine adäquate Gewebeoxygenierung zu ermöglichen.

## Therapie und Therapieziele

### Chirurgische Therapie

In der chirurgischen Behandlung des SHT steht die **Hämatomevakuation**

Hämatomevakuation

im Vordergrund. Die Größe des operativen Zugangs richtet sich hierbei nach der Art der Blutung. Beim cSDH reicht primär eine Bohrlochtrepanation, bei EDH, intrazerebraler Blutung und aSDH bedarf es einer Kraniotomie bis hin zu einer Kraniektomie zur Hämatomentleerung. Aktuelle Daten scheinen die Notwendigkeit einer dekompressiven Hemikraniektomie jedoch infrage zu stellen [52].

Neben der reinen Hämatomausräumung kann eine Kraniotomie auch zur **Hirndrucksenkung**

Hirndrucksenkung

dienen. Hier ist jedoch ein **strenger Eskalationsansatz**

Strenger Eskalationsansatz

im Sinne eines Stufenschemas zu verfolgen, der von nichtinvasiven Methoden bis hin zu operativen Verfahren reicht [17, 51].

Studien wie DECRA und RESCUE-ICP haben die Wirksamkeit der späten **dekompressiven Hemikraniektomie**

Dekompressive Hemikraniektomie

bei Patienten mit sekundär refraktärem Hirndruck (> 20 mm Hg für mehr als 15 min) untersucht. Die Ergebnisse zeigen, dass eine **frühzeitige Entlastung**

Frühzeitige Entlastung

innerhalb der ersten 72 h nach dem Ereignis keine signifikanten Vorteile bringt und die Mortalität sogar erhöhen kann. Im Gegensatz dazu ließ sich zeigen, dass eine **spätere Entlastung**

Spätere Entlastung

nach 72 h bis 10 Tagen zu einer verbesserten klinischen Erholung führt und die Mortalitätsrate senkt. Diese Erkenntnisse legen nahe, dass eine sorgfältige Abwägung des richtigen Zeitpunkts für die Durchführung der dekompressiven Hemikraniektomie von entscheidender Bedeutung ist, um die bestmöglichen Ergebnisse für die Patient:innen zu erzielen [53]. Trotz teilweise diskussionswürdiger Ein- und Ausschlusskriterien sollte die dekompressive Hemikraniektomie bei refraktärem ICP-Anstieg zum Armamentarium gegen lang anhaltenden, malignen Hirndruck gehören. Allerdings muss der mögliche klinische Gewinn das operative Risiko übertreffen [54].

#### Merke

Die dekompressive Hemikraniektomie dient zur Reduktion des ICP und soll eine verbesserte zerebrale Perfusion ermöglichen.

### Neuromonitoring

Neben der Bildgebung kommt dem **multimodalen Neuromonitoring**

Multimodales Neuromonitoring

ein zunehmender Stellenwert im Management und auch in der Prognosestellung beim SHT zu. Kontinuierliches zerebrales multimodales Neuromonitoring verbessert nachweislich die Behandlung kritisch kranker neurochirurgischer Patient:innen, insbesondere nach schwerem SHT [50, 55]. Bei diesen Patient:innen ist häufiger ein zerebrales Neuromonitoring für bis zu 2–3 Wochen erforderlich und empfohlen, da ihr neurologischer Zustand aufgrund einer gestörten Vigilanz bzw. Intubation nicht beurteilbar ist [17]. Das Neuromonitoring wird entweder im Zuge der Hämatomausräumung oder – vor allem bei DAI – in einer gesonderten Operation angelegt. Standardmäßig und empfohlenermaßen soll der ICP gemessen werden, Parameter wie die Sauerstoffgewebssättigung sowie Stoffwechselvorgänge gehören noch nicht zur Routine.

#### Intrakranieller Druck

Eine ICP-Sonde ist bei schwerem SHT sowie einem pathologischen CT-Befund indiziert. Ebenso besteht eine Indikation bei Patient:innen mit schwerem SHT, aber normalem CT-Befund, wenn das Patientenalter über 40 Jahre ist, einseitige oder beidseitige Strecksynergismen bestehen und/oder ein systolischer Blutdruck unter 90 mm Hg besteht [17].

Es werden zwei Messmethoden unterschieden, entweder mittels einer rein **parenchymatösen Hirndrucksonde**

Parenchymatöse Hirndrucksonde

oder mittels einer **externen Ventrikeldrainage**

Externe Ventrikeldrainage.

Mit Letzterer kann nicht nur der Druck gemessen, sondern auch zeitgleich ein erhöhter Hirndruck mittels Liquordrainage therapiert werden. Beide Techniken zeichnen sich durch eine geringe Komplikationsrate aus. Hinsichtlich der Hirndrucktherapie empfiehlt sich das kontinuierliche Ablassen von Liquor im Gegensatz zur intermittierenden Öffnung. Zur Hirndruckmessung sind kurze Verschlusszeiten notwendig [56].

Bei anhaltend hohem ICP über 22 mm Hg mit konsekutiver Verringerung des CPP über einen Zeitraum von mehr als 15 min kann es zu einer Schädigung der Neurone kommen, weshalb als Cut-off zur Behandlung eines erhöhten ICP aktuell 22 mm Hg empfohlen werden [56].

#### Parenchymatöse Sauerstoffmessung

Als Erweiterung des Neuromonitorings kann die **Sauerstoffgewebssättigung**

Sauerstoffgewebssättigung

(P_ti_O_2_) bzw. der **Sauerstoffpartialdruck**

Sauerstoffpartialdruck

gemessen werden. Hierzu wird eine Sonde in das Parenchym eingebracht, idealerweise in Gewebearealen, welche von einer Minderperfusion betroffen sein können. Ein Partialdruck über 20 mm Hg sollte angestrebt werden. Eine Kombination von ICP und P_ti_O_2_ in der Therapie scheint das Outcome zu verbessern [56].

#### Mikrodialyse

Im Rahmen der Mikrodialyse können über eine parenchymatöse Sonde verschiedene **Stoffwechselprodukte**

Stoffwechselprodukte

analysiert werden, beispielsweise Laktat, Pyruvat und Glukose. Über diese Werte können metabolische Veränderungen detektiert werden. Aktuell wird die Mikrodialyse jedoch primär zu wissenschaftlichen Zwecken verwendet, Niederschlag im klinischen Alltag hat sie noch nicht gefunden.

### Nichtchirurgische/medikamentöse Therapie

#### Hirndrucktherapie

Als erster Schritt des Stufenkonzepts zur Senkung des ICP werden unter anderem folgende Maßnahmen angewendet:

- Erhöhung des Oberkörpers um 30 Grad zur Verbesserung des venösen Abflusses
- Aufrechterhaltung normaler Blutdruck- und CPP-Werte
- Behandlung möglicher epileptogener Ursachen
- Schmerz- und Laxanzientherapie

In einigen Fällen versagt die Erstlinientherapie, woraufhin Zweitlinienverfahren zur Anwendung kommen. Diese umfassen

- die Verwendung hyperosmolarer Substanzen,
- kurzfristige leichte Hyperventilation,
- vertiefte Sedierung,
- die Applikation von Vasopressoren und
- neuromuskuläre Blockade.

In aktuellen Studien überwiegt der positive Effekt von **hypertoner Kochsalzlösung**

Hypertone Kochsalzlösung

gegenüber **Mannitol**

Mannitol

[56]. Allerdings hat die Gabe von hyperosmolaren Medikamenten keinen signifikanten Einfluss auf die klinische Erholung und Mortalität [57]. Steroide haben beim SHT keine Berechtigung und sind sogar mit einer erhöhten Mortalität assoziiert.

Wenn trotz aller Bemühungen der ICP weiterhin erhöht bleibt, müssen Drittlinienverfahren in Betracht gezogen werden. Hierbei handelt es sich um fortgeschrittene Maßnahmen, unter anderem die Verabreichung von Medikamenten wie **Pentobarbital**

Pentobarbital

oder **Thiopental**

Thiopental,

um ein Koma zu induzieren, und die **therapeutische Hypothermie**

Therapeutische Hypothermie,

um den Stoffwechsel zu verlangsamen und die Schädigung des Gehirns zu reduzieren. Entsprechende Studien konnten jedoch die Sinnhaftigkeit der Hypothermie nicht belegen. Im Kontext der Drittlinienverfahren muss auch die dekompressive Kraniektomie gesehen werden (siehe Abschnitt „Chirurgische Therapie“).

##### Merke

Hypertone Kochsalzlösung ist eine hyperosmolare Lösung, mit der ein erhöhter ICP behandelt werden kann.

#### Epileptische Anfälle

Eine traumaassoziierte Epilepsie kann sich als **frühe Form**

Frühe Form,

die innerhalb von 7 Tagen nach dem Trauma auftritt, und als **späte Form**

Späte Form,

die nach mehr als 7 Tagen auftritt, manifestieren. Eine sorgfältige Literaturrecherche von Fordington et al. [58] hat gezeigt, dass viele Studien zur Bewertung der Wirksamkeit von prophylaktischen Antiepileptika beim SHT mangelhaft sind. Im Hinblick auf die Substanzen, welche verabreicht werden können, ergaben Studien keine signifikanten Vorteile für Valproinsäure, Levetiracetam oder Phenytoin. Auch beim frühen posttraumatischen Status epilepticus konnte kein Unterschied zwischen Levetiracetam und Phenytoin gefunden werden. Basierend auf diesen Erkenntnissen empfiehlt die BTF derzeit keine antiepileptische Prophylaxe bei SHT [17].

##### Merke

Eine antiepileptische Prophylaxe wird derzeit beim SHT nicht empfohlen.

#### Gerinnung

Im Rahmen der CRASH-3-Studie wurde der Einsatz von **Tranexamsäure**

Tranexamsäure

(TXA) bei Patient:innen mit SHT untersucht. Die Ergebnisse dieser Studie belegen, dass beim leichten und moderaten SHT die frühzeitige Verabreichung von TXA innerhalb von 3 h nach dem Trauma das Mortalitätsrisiko reduziert, ohne dass signifikante unerwünschte Ereignisse auftreten. Bei Patienten mit schwerem SHT konnte hingegen kein signifikanter Effekt beobachtet werden [59].

## Fazit für die Praxis

- Das Schädel-Hirn-Trauma (SHT) ist ein Erkrankungskomplex, der junge Patient:innen, aber auch immer mehr ältere Patient:innen betrifft. Ursächlich sind primär Stürze, gefolgt von Verkehrs- und Freizeitunfällen.
- Es lassen sich drei SHT-Schwergrade unterscheiden, die anhand des Glasgow-Coma-Scale(GCS)-Scores eingeteilt werden. Ein GCS-Score von 13 bis 15 beschreibt ein leichtes SHT, ein Score von 9 bis 12 ein moderates SHT und ein Wert von 3 bis 8 ein schweres SHT.
- Raumfordernde intrakranielle Blutungen müssen häufig operativ entleert werden. Je nach Lokalisation der Blutung bzw. deren Alter kann das chirurgische Vorgehen variieren.
- Ein intrakranieller Druck (ICP) über 22 mm Hg gilt als Behandlungsindikation, wobei hier ein eskalierendes Stufenschema angewendet werden soll. Bei der Therapie mit hyperosmolaren Lösungen ist hypertone Kochsalzlösung gegenüber Mannitol zu bevorzugen.
- Als Folge des primären Traumas kommt es zu einer pathophysiologischen Kaskade, deren Behandlung das primäre Ziel ist. In dieser Phase muss neben dem ICP auch auf die zerebrale Perfusion geachtet werden.

## CME-Fragebogen

Ein 45-jähriger Mann stürzt beim Bergsteigen ca. 5 m in die Tiefe und erleidet ein schweres Schädel-Hirn-Trauma. Er wird vom Notarzt intubiert und in den Schockraum gebracht, wo eine bildgebende Abklärung des Schädels mittels Computertomographie (CT) erfolgt. Dort zeigt sich eine hyperdense, sichelförmige Blutung unterhalb der Kalotte, siehe Abb. 8. Was ist die Diagnose?

Akutes Subduralhämatom

Epidurales Hämatom

Chronisches Subduralhämatom

Intrazerebrale Blutung

Subarachnoidalblutung

Eine 25-jährige Patientin wird nach einem Unfall in der Notaufnahme eingeliefert und präsentiert sich neurologisch wie folgt: a. Öffnet die Augen auf Schmerzreiz. b. Gibt nur einzelne Worte von sich, jedoch ohne Zusammenhang. c. Führt gezielte Bewegungen bei Schmerzreiz aus. Welchen Glasgow Coma Score hat die Patientin?

10

15

8

13

12

Bei welchem Blutungstyp kommt es zu einer Blutung zwischen Dura und Periost des inneren Schädels und wird meist durch die Suturen begrenzt?

Epiduralhämatom

Diffuser axonaler Schaden

Akutes Subduralhämatom

Hämorrhagischer Schlaganfall

Intrazerebrale Blutung

Ein 84-jähriger Patient wird in die Notaufnahme eingeliefert, da er seit 2 Tagen deutliche Probleme beim Sprechen und eine Schwäche im Bereich der rechten Körperhälfte hat. Der begleitende Sohn des Patienten gibt an, dass sein Vater zu Hause immer wieder stürze und sich den Kopf anschlage. Sie veranlassen eine Computertomographie und sehen eine hypodense, sichelförmige Blutung über der linken Hemisphäre. Wie lautet die Diagnose?

Chronisches Subduralhämatom

Epidurales Hämatom

Akutes Subduralhämatom

Hämorrhagischer Schlaganfall

Intrazerebrales Hämatom

Was ist die häufigste Ursache für Schädel-Hirn-Traumata?

Spaziergänge

Stürze

Verkehrsunfälle

Fahrradunfälle

Schussverletzungen

Welche Bildgebung wird für die initiale Untersuchung von traumatischen Kopfverletzungen im Notfall empfohlen?

Magnetresonanztomographie

Positronenemissionstomographie

Computertomographie

Röntgen

Ultraschall

Eine traumaassoziierte Epilepsie kann sich als frühe (innerhalb von 7 Tagen nach dem Trauma) oder späte Form (nach mehr als 7 Tagen) manifestieren. Welche prophylaktische Antiepileptikabehandlung wird von der Brain Trauma Foundation empfohlen?

Valproinsäure

Levetiracetam

Phenytoin

Keine

Eine Kombinationstherapie

Welche der angeführten Indikationen ist richtig betreffend die operative Entlastung eines akuten Subduralhämatoms?

Mittellinienverschiebung kleiner als 5 mm und Glasgow-Coma-Scale(GCS)-Score > 12

Hämatomdicke > 5 mm, unabhängig vom Glasgow-Coma-Scale(GCS)-Score

Intrakranieller Druck (ICP) konstant > 15 mm Hg, unabhängig von der Dicke des Hämatoms sowie der Mittellinienverschiebung

Jedes Hämatom, das mit einer Fraktur einhergeht

Hämatomdicke > 10 mm, unabhängig vom Glasgow-Coma-Scale(GCS)-Score

Ein 35-jähriger Mann wird nach einem Autounfall in die Notaufnahme gebracht. Er ist bei Bewusstsein, aber desorientiert und zeigt eine langsame und unspezifische Reaktion auf verbale Aufforderungen. Die Pupillen sind beidseitig erweitert und reagieren träge auf Licht. Welche Erstmaßnahme ist dringend notwendig?

Gabe von Schmerzmitteln

Computertomographie-Scan des Schädels

Abdominelle Abklärung

Gabe von Antibiotika

Gabe von Antiemetika

Eine 45-jährige Frau wird mit einem Schädel-Hirn-Trauma in die Notaufnahme eingeliefert. Die klinische Untersuchung zeigt einen Glasgow-Coma-Scale(GCS)-Score von 7 und die computertomographische Untersuchung zeigt eine schwere traumatische Subarachnoidalblutung und den Verdacht auf einen diffusen axonalen Schaden. Die Mittellinie ist nicht verschoben und es ist kein weiteres Hämatom abgrenzbar. Welche therapeutische Option ist in diesem Fall am wahrscheinlichsten indiziert?

Osteoplastische Kraniotomie

Osteoklastische Kraniotomie

Keine Operation, Gabe von Steroiden

Anlage eines multimodalen Neuromonitorings

Keine Operation, Observation auf der Intensivstation
